# Longitudinal investigation of carriage rates and genotypes of toxigenic *Clostridium difficile* in hepatic cirrhosis patients

**DOI:** 10.1017/S0950268819000554

**Published:** 2019-03-28

**Authors:** Yunbo Chen, Hongqin Gu, Tao lv, Dong Yan, Qiaomai Xu, Silan Gu, Ping Shen, Jiazheng Quan, Yunhui Fang, Lifeng Chen, Guangyong Ye, Lanjuan Li

**Affiliations:** 1 State Key Laboratory for Diagnosis and Treatment of Infectious Diseases, Collaborative Innovation Center for Diagnosis and Treatment of Infectious Diseases, The First Affiliated Hospital, School of Medicine, Zhejiang University, Hangzhou, Zhejiang, China; 2 Clinical Laboratory, Hangzhou Third Hospital, Hangzhou, Zhejiang, China; 3 Medical Engineering Department, The First Affiliated Hospital, School of Medicine, Zhejiang University, Hangzhou, Zhejiang, China; 4 Women's Hospital, School of Medicine, Zhejiang University, Hangzhou, Zhejiang, China

**Keywords:** Carriage, *Clostridium difficile*, genotype

## Abstract

Toxigenic *Clostridium difficile* (*C. difficile*) carriers represent an important source in the transmission of *C. difficile* infection (CDI) during hospitalisation, but its prevalence and mode in patients with hepatic cirrhosis are not well established. We investigated longitudinal changes in carriage rates and strain types of toxigenic *C. difficile* from admission to discharge among hepatic cirrhosis patients. Toxigenic *C. difficile* was detected in 104 (19.8%) of 526 hepatic cirrhosis patients on admission, and the carriage status changed in a portion of patients during hospitalisation. Approximately 56% (58/104) of patients lost the colonisation during their hospital stay. Among the remaining 48 patients who remained positive for toxigenic *C. difficile*, the numbers of patients who were positive at one, two, three and four isolations were 10 (55.6%), three (16.7%), two (11.1%) and three (16.7%), respectively. Twenty-eight patients retained a particular monophyletic strain at multiple isolations. The genotype most frequently identified was the same as that frequently identified in symptomatic CDI patients. A total of 25% (26/104) of patients were diagnosed with CDI during their hospital stay. **Conclusions:** Colonisation with toxigenic *C. difficile* strains occurs frequently in cirrhosis patients and is a risk factor for CDI.

## Introduction

*Clostridium difficile* (*C. difficile*) infection (CDI) is one of leading causes of mortality in the developed world [1] and estimated to be responsible for 10–20% of antibiotic-associated diarrhoea cases including all cases of pseudomembranous colitis [2], resulting in an estimated medical cost of €3 billion per annum among the EU states [3] and $1.5 billion per annum in the USA [4]. It has been accepted that this organism spreads nosocomially and causes outbreaks of CDI in various clinical settings [2]. Infected or colonised patients and contaminated environments constituted potential sources of *C. difficile* [5] in hospital settings. Asymptomatic carriers shed spores into the environment to a lesser extent than CDI patients [6], but because *C. difficile* colonisation is fivefold to 10-fold more common than symptomatic infection [7], they serve as an important reservoir for nosocomial transmission. Nearly 30 years ago, Clabots *et al*. found that most episodes of nosocomial acquisition of CDI in a study ward were epidemiologically linked to transmission from asymptomatic new admissions [8]. This link was more recently supported by a Canadian study, in which the isolation of *C. difficile*-colonised patients was accompanied by a significantly reduced incidence of hospital-acquired CDI in recent years [9]. It was also reported that asymptomatic colonisation increases the risk of subsequent clinical disease [10]. Approximately 5–15% of patients newly admitted to hospitals carry *C. difficile* in their faeces [10, 11], and it has been reported that one-sixth to one-third of the carriers may develop symptoms [12].

However, there have been no reports of associations between noted involvement of asymptomatic carriage in the transmission of toxigenic *C. difficile* in healthcare facilities and corresponding practice to block the transmission [13], as they have not been a focus of CDI control measures [14]. It is noteworthy that there was a different impression that asymptomatic colonisation with *C. difficile* was associated with a decreased risk of diarrhoea [15].

The carriage and transmission frequencies of *C. difficile* have been studied in an elderly population living in long-term care facilities (LTCFs) [16], as well as in healthy adults aged up to 65 years (median age, 22 years) in Japan [17], many of whom were college students. To the best of our knowledge, there have been no studies of carriage among patients with a specific disease. For example, there are no reports on liver cirrhotic patients, who are more prone to acquire CDI as a result of disturbed microbiota in the gut, increased hospital visits and antibiotic usage. In this study, we investigated the asymptomatic carriage and genotype of *C. difficile* in these patients using stool specimens collected from admission to discharge. We determined the frequency of asymptomatic toxigenic *C. difficile* carriage at admission and at different time points after hospitalisation. We also investigated whether the strains isolated from these patients represented current endemic *C. difficile* genotypes in China.

## Materials and methods

### Study design

During a 6-month period in 2015 (1 May to 31 October), we conducted a cohort study of all patients who provided consent in the Infectious Disease Department of the First Affiliated Hospital, Zhejiang University. On average, there were 168 admissions per ward per month, including 34 admissions that stayed for more than 48 h. Cirrhosis was diagnosed based on previous liver biopsy results, clinical evidence of previous decompensation and laboratory tests, endoscopy and radiological imaging of portal hypertension and/or liver nodularity. Patients were excluded if they: had hepatic carcinoma or other malignancies, had diarrhoea on admission, were discharged within 2 days, were transferred from other wards to the participating wards and were readmitted during this period. Stool samples were collected within 48 h of admission and then weekly during hospitalisation until discharge or diagnosis with CDI.

Written informed consent was obtained from each patient. The study protocols were approved by the Ethical Committee of the First Affiliated Hospital, Zhejiang University School of Medicine.

### Definitions

Asymptomatic carriage was defined as positivity for toxigenic *C. difficile* without symptoms of diarrhoea at the time of stool collection [18] or in the first 48 h of admission. If identified in a negative patient after 48 h, nosocomial acquisition was considered. CDI was defined by diarrhoea episodes (⩾3 unformed stools in 24 h) occurring at least 48 h after admission, diagnosis with the two-stage algorithms, and negative results for other diarrhoea-causing pathogens including *Salmonella* spp., *Shigella* spp., *Vibrio* spp., *Staphylococcus aureus* and *Escherichia coli*.

When the patient was documented with *C. difficile* diarrhoea, caregivers and others were advised to exercise contact precautions, including using gloves, wearing gowns and using chlorhexidine for hand hygiene.

### Isolation of *C. difficile* from faecal samples and diagnosis of CDI

The stool samples, including admission and follow-up during hospitalisation, were cultured on cycloserine-cefoxitin-fructose agar in an atmosphere composed of 80% N_2_, 10% H_2_ and 10% CO_2_ at 37 °C for 48 h. A maximum of five colonies was picked from each *C. difficile*-positive plate. Colonies with typical morphology and odour were confirmed as *C. difficile* by matrix-assisted laser desorption-ionisation mass spectrometry (MALDI-TOF MS, Flexcontrol3.3-microflex).

The two-stage algorithms were used to confirm the microbiological evidence of toxin-producing *C. difficile* in stools. Only unformed or watery stool samples were detected for *C. difficile* toxins (Bristol Stool Chart types 5–7) [19]. The protocol of the two-stage algorithms, including glutamate dehydrogenase and toxin A and B detection by enzyme immunoassay (EIA) (Vidas; bio-Mérieux, Marcy-l'Etoile, France), was described previously [20].

### DNA extraction and detection of toxigenic genes

The isolates of *C. difficile* were grown on blood agar incubated anaerobically for 48 h. Genomic DNA from the two isolates was extracted using the QIAamp DNA Mini Kit (Qiagen, Hilden, Germany). *TcdA* and *tcdB* genes were detected by PCR as described previously [21]. Both binary toxin genes, *cdtA* and *cdtB*, were detected as described by Stubbs *et al*. [22].

### Multi-locus sequence typing

Multi-locus sequence typing (MLST) was performed on all toxigenic strains according to the previously described protocols [23]. The sequence type (ST) was determined according to a combination of alleles identified by comparing the obtained sequences with sequences available in the *C. difficile* MLST database available at: http://pubmlst.org/cdifficile/.

## Results

**Fig. 1.**
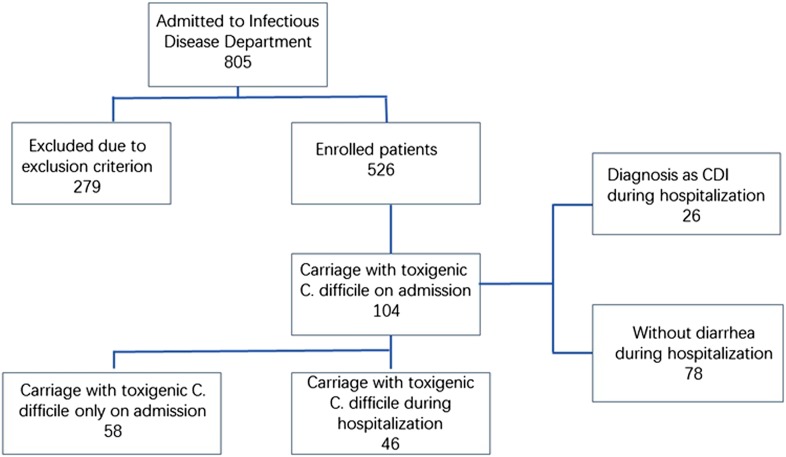
Flow chart of patient recruitment and selection.

A total of 805 patients were admitted during the study period, and 279 of them were excluded. Included in the evaluation were 1056 faeces samples from 526 patients, of which 104 (19.8%) were positive for toxigenic *C. difficile* upon admission (Fig. 1). Follow-up was performed on these 104 patients weekly until they were discharged or diagnosed with CDI. Among these 104 patients, 44.2% (46/104) of the patients had at least one toxigenic *C. difficile*-positive as least one time during their hospitalisation and the remainder had only positive at admission. Sixteen ST types were identified among the 104 isolates from admission samples (Table 1). The top four types were ST-54 (18.5%, 19/104), ST-35 (15.4%, 16/104), ST-3 (21.2%, 22/104) and ST-37 (14.4%, 15/104). There were 76 isolates and 17 ST types identified during the follow-up. The top four types were ST-54 (26.3%, 20/76), ST-3 (17.1%, 13/76), ST-35 (11.8%, 9/76) and ST-37 (6.6%, 5/76). ST-15, ST-109, ST-220 and ST-294 were identified on admission and became negative afterwards, while new STs including ST-2, ST-26, ST-48, ST-55 and ST-129 emerged. Continuous colonisation with the same STs was detected in 28 patients (26.9%), while different STs were identified in 18 patients (17.3%) over the course of hospitalisation. Ten STs detected on admission were changed during hospitalisation. ST-35 and ST-37 were most frequently changed to other STs (25% and 26.7%, respectively). Among the 46 patients who remained positive after admission, the percentages who were positive for two, three, four, or more isolations were 29.8% (31/104), 5.8% (6/104) and 8.7% (9/104), respectively. No *C. difficile* NAP1/BI/027/ST1 or 078 isolates were identified in this study, and no specimens contained two or more different toxin types.

**Table 1.** Positive isolations and sequence types from 104 patients with *C. difficile* colonisation and/or infection

No of Patients	Gender	Age	Isolated on admission	Isolated in week 2	Isolated in week 3	Isolated in week 4	Isolated in week 5	Isolated in week 6	Isolated in week 7	Isolated in week 8	Diagnosed with CDI
P1	Female	65	ST-3	ST-3							
P2	Male	51	ST-3								
P3	Male	49	ST-3								
P4	Male	45	ST-3								
P5	Male	47	ST-3								
P6	Male	54	ST-3								
P7	Male	49	ST-3								
P8	Male	56	ST-3								
P9	Male	61	ST-3								
P10	Male	75	ST-3	ST-3							
P11	Male	43	ST-3	ST-3						ST-3	
P12	Female	76	ST-3								
P13	Female	52	ST-3			ST-3					
P14	Male	22	ST-3								
P15	Male	59	ST-3								
P16	Male	75	ST-3	ST-3	ST-3	ST-3					CDI
P17	Male	39	ST-3								
P18	Male	46	ST-3	ST-3							
P19	Male	35	ST-3								
P20	Male	30	ST-3	ST-26							
P21	Male	56	ST-3								
P22	Male	55	ST-3	ST-55							
P23	Male	59	ST-6		ST-6						CDI
P24	Male	51	ST-8								
P25	Male	65	ST-8	ST-8							CDI
P26	Male	53	ST-8								
P27	Male	32	ST-8				ST-54				
P28	Male	43	ST-8	ST-8							CDI
P29	Female	56	ST-8								
P30	Female	51	ST-14	ST-14							CDI
P31	Male	54	ST-14								
P32	Male	52	ST-14								
P33	Male	41	ST-14	ST-14							CDI
P34	Male	75	ST-14								
P35	Female	63	ST-14								
P36	Male	59	ST-14								
P37	Female	56	ST-15	ST-15	ST-15	ST-35					CDI
P38	Male	60	ST-29	ST-29		ST-29	ST-29				CDI
P39	Male	39	ST-34		ST-34						CDI
P40	Female	61	ST-35								
P41	Male	42	ST-35	ST-35							CDI
P42	Male	42	ST-35			ST-129	ST-129	ST-129			
P43	Male	49	ST-35								
P44	Male	54	ST-35		ST-35	ST-37					CDI
P45	Female	58	ST-35	ST-35							CDI
P46	Female	70	ST-35								
P47	Female	49	ST-35		ST-35		ST-37	ST-37	ST-37		CDI
P48	Male	64	ST-35		ST-48						
P49	Male	45	ST-35								
P50	Female	62	ST-35								
P51	Male	66	ST-35	ST-35	ST-35						CDI
P52	Male	54	ST-35								
P53	Male	47	ST-35								
P54	Male	63	ST-35								
P55	Male	71	ST-35								
P56	Female	70	ST-37								
P57	Female	90	ST-37								
P58	Male	49	ST-37	ST-2							
P59	Male	26	ST-37	ST-2							
P60	Male	66	ST-37								
P61	Male	48	ST-37	ST-2							
P62	Female	55	ST-37		ST-3	ST-3					
P63	Male	50	ST-37								
P64	Male	36	ST-37								
P65	Male	54	ST-37								
P66	Male	60	ST-37								
P67	Male	68	ST-37	ST-37							CDI
P68	Female	47	ST-37								
P69	Male	68	ST-37								
P70	Male	56	ST-37								
P71	Male	61	ST-39								
P72	Male	44	ST-39								
P73	Female	67	ST-39								
P74	Male	65	ST-39	ST-39	ST-39	ST-39					CDI
P75	Male	72	ST-39		ST-54						
P76	Male	46	ST-39								
P77	Male	42	ST-54	ST-54							CDI
P78	Male	66	ST-54				ST-54				
P79	Male	52	ST-54								
P80	Male	45	ST-54								
P81	Male	66	ST-54	ST-54							CDI
P82	Female	72	ST-54	ST-35							
P83	Female	54	ST-54								
P84	Male	54	ST-54	ST-54	ST-54	ST-54	ST-54		ST-54	ST-54	CDI
P85	Male	40	ST-54		ST-54	ST-54		ST-54	ST-54		CDI
P86	Female	69	ST-54								
P87	Male	58	ST-54								
P88	Male	72	ST-54								
P89	Male	47	ST-54	ST-54	ST-54						CDI
P90	Male	54	ST-54	ST 54							
P91	Male	44	ST-54	ST-54	ST 54						CDI
P92	Male	49	ST-54								
P93	Male	42	ST-54								
P94	Male	74	ST-54								
P95	Male	39	ST-54								
P96	Male	43	ST-81			ST-8					CDI
P97	Male	59	ST-81								
P98	Male	46	ST-81		ST-8						CDI
P99	Male	47	ST-102	ST-3							
P100	Female	59	ST-102								
P101	Female	56	ST-102	ST-102	ST-102	ST-102	ST-102				CDI
P102	Female	66	ST-294	ST-35							CDI
P103	Male	48	ST-109								
P104	Male	45	ST-220	ST-3							

CDI occurred in 25% (26/104) of patients during their hospital stay. The percentages of patients who had two, three and four positive isolations before diagnosis with CDI were 53.8% (14/26), 15.4% (4/26) and 15.4% (4/26), respectively. Three patients remained positive for five isolations and one for seven isolations. These four patients were diagnosed with CDI in the last sample. The time between detection of colonised *C. difficile* and CDI diagnosis was 1–2 weeks.

## Discussion

One uniqueness in this study is the availability of first stool samples taken at or near admission and then the collection of weekly samples afterwards, which allowed us to study *C. difficile* colonisation and infection kinetics. Toxigenic *C. difficile* was detected in 19.8% of hepatic cirrhosis patients on admission. Different colonisation rates have been reported by different studies [10], and several explanations are possible. (1) Different detection methods were employed. Nucleic acid amplification tests were the most sensitive in some studies [24], while toxin EIAs were suboptimal in sensitivity. In this study, stool sample cultures, which are more sensitive than swab cultures, were performed [25]. (2) Various risk factors are associated with asymptomatic *C. difficile* carriage including age, admission from another healthcare facility, overnight hospitalisation within the prior 90 days and exposure to antibiotics in the 90 days prior to admission [26]. Our cirrhosis patients may have had these risk factors for toxigenic *C. difficile* carriage, leading to a higher carriage rate compared with those previously reported [27].

ST-54, ST-35, ST-3 and ST-37 were the most prevalent among admission samples, and these STs were reported as the most common STs causing *C. difficile* infection in China [28–30]. In this study, 25% (26/104) of these patients developed CDI during their hospital stay, and ST-54 and ST-35 were the dominant types identified in these CDI patients [31]. These results were consistent with those of a report that the asymptomatic *C. difficile* carriage rate is similar to the symptomatic positivity rate, implying that CDI in the majority of symptomatic patients was likely derived from *C. difficile* colonisation [15], which represents a significant infection risk. All these reports support the hypothesis that the admission of asymptomatic *C. difficile* colonised patients contributes to sustained *C. difficile* transmission within a ward [32].

In this study, nearly 60% of the patients were positive only in admission samples, suggesting a transient colonisation. As reported in a 6-month follow-up of 18 colonised healthy students, 10 (56%) of them lost the colonisation [17]. Only 46 patients (40%) were positive for multiple isolations in this cohort, comparing eight (44%) of 18 colonised more than once, of whom three (38%) harboured the same strain previously reported [17]. However, there were no reports of colonisation during the follow-up of the hospitalised population. There were patients who showed continuous colonisation of toxigenic *C. difficile* after admission, and thus, hospitalisation may be a risk factor for colonisation in this study. There were three patients who were *C. difficile*-positive three or more times, and the *C. difficile* isolates from each of the three patients were different from the first isolates, but for all isolates, the same isolates were detected in at least two consecutive occasions. These findings suggest that there is a marked variation in the duration of the colonised state and the environment may have been contaminated. In an investigation of repeated exposure from the environment or other colonised individuals, *C. difficile* strains with the same STs were isolated from 28 patients (26.9%) during their hospital stay. These results suggest that cross-transmission of *C. difficile* may be relatively common among colonised individuals, or *C. difficile* may spread from a common source in the work environment.

Our previous study and other reports have shown that carriers of *C. difficile* are significantly more likely to develop diarrhoea during hospitalisation than non-carriers [31, 33]. Asymptomatic carriage identified at admission was a decisive risk factor for symptomatic infection during hospital stay, accounting for about 25% of the patients (26 of 104) who developed CDI during the study. Thus, screening for toxigenic *C. difficile* carriage at admission could predict the likelihood of a later symptomatic CDI, as described in previous studies [10, 14].

Another important finding was with an increasing number of positive isolations, the risk for CDI increased. In this study, of 15 patients who were positive for multiple isolations, 12 developed CDI. This study showed that different types of *C. difficile* influenced the infection. It is important to screen the colonisation of toxigenic *C. difficile* when patients were admitted, especially for those patients who have a higher risk for infection.

On average, it takes 5 days for colonised patients to develop symptoms [32, 34]. In this cohort, symptom development took 1–2 weeks, except in one patient who was diagnosed with CDI after 2 weeks. This was similar to other reports [27, 35].

We note some limitations in this study. First, we tried to collect samples from patients within 2 days after admission. However, some patients were excluded because they had no stools within 2 days, reducing the number of patients with bias for the colonisation rate. Second, we did not take into consideration the therapy, especially antibiotic treatment during hospital stay, which is expected to have an impact on the colonisation of *C. difficile*. Third, we did not re-examine the patients who were negative for *C. difficile* at admission, and these patients may colonise *C. difficile* during their hospitalisation. Finally, we cannot be certain that the individuals who were colonised with toxigenic *C. difficile* on admission to the LTCF acquired colonisation during their hospital stay; it was possible that they carried *C. difficile* at the time of admission to the hospital.

## Conclusions

Toxigenic *C. difficile* was detected in 104 (19.8%) of 526 hepatic cirrhosis patients on admission. Moreover, the carriage status changed in a portion of patients, whereas 55.8% (58//104) of patients lost the colonisation of toxigenic *C. difficile* during their hospital stay. Among the remaining 48 patients who remained positive for toxigenic *C. difficile*, 25% (26/104) were diagnosed with CDI during hospital stay. This study highlights the importance of identifying asymptomatic *C. difficile* carriers among hepatic cirrhosis patients on admission.
